# Living Near Contamination: The Impacts on Personal Well-Being

**DOI:** 10.1007/s11482-025-10456-8

**Published:** 2025-06-12

**Authors:** Sara Burcham, Wei-Wen Hsu, Jack Rubinstein, Sharon L. Larson, Susan M. Pinney

**Affiliations:** 1 Department of Environmental and Public Health Sciences, Division of Epidemiology, University of Cincinnati College of Medicine, Cincinnati, USA; 2 Department of Environmental and Public Health Sciences, Division of Biostatistics and Bioinformatics, University of Cincinnati College of Medicine, 160 Panzeca Way, Cincinnati, OH 45267 USA; 3 Department of Internal Medicine, College of Medicine, University of Cincinnati, Cincinnati, USA; 4 Population Health Sciences, Thomas Jefferson University, Philadelphia, PA USA

**Keywords:** Well-being, Environmental contamination, Health risk appraisal, Life satisfaction, Residential proximity, Epidemiology

## Abstract

The impact of residential proximity (RP) to EPA-designated Superfund sites on personal well-being remains understudied. This study evaluated the well-being of residents living near a former uranium processing facility in Fernald, Ohio, now a Superfund site, which emitted significant amounts of radioactive hazardous waste. Baseline data were collected from 7,957 participants who voluntarily enrolled in the Fernald Community Cohort. Participants completed the Health Risk Appraisal (HRA), which assessed four well-being indicators: Life Satisfaction, Life in Control, Life Perspective, and Overall Physical Health. Separate multivariable ordinal logistic regression models were used to evaluate the cross-sectional relationship between RP and well-being outcomes. The results indicated that residents living within a 1-mile radius of the Superfund site had 38% greater odds of reporting lower Life Satisfaction compared to those residing 4–5 miles away [OR 1.38, 95% CI: 1.17, 1.64]. In the fully adjusted models, other significant risk factors emerged. Participants who reported two or more recent misfortunes in the past year were more likely to report a negative Life Perspective [OR 2.79, 95% CI: 2.36, 3.32]. Additionally, those with a weakened social ties had significantly higher odds of reporting diminished Life Satisfaction [OR 5.16, 95% CI: 4.33, 6.16]. This study identified RP to a Superfund site and psychosocial characteristics as significant predictors of well-being. Given the global prevalence of contaminated sites, particularly in socioeconomically disadvantaged regions, further investigation into their impact on local communities is essential.

**Supplementary Information:**

The online version contains supplementary material available at 10.1007/s11482-025-10456-8.

## Introduction

An estimated one in four Americans live within three miles of a U.S. Environmental Protection Agency (EPA) designated “Superfund” site, yet research on the impact of these sites on personal well-being is sparse (U.S. Environmental Protection Agency & Office of Land and Emergency Management, 2023). The presence of a Superfund site in a community can cause psychological stress as residents evaluate the riskiness of the environmental hazard (Cortés et al., 2021). Communities surrounding contaminated sites experience unique psychosocial challenges, including uncertainty about the level of exposure due to the often invisible nature of toxic substances. Additionally, the long lag time between exposure and the onset of disease complicates the ability to link past exposures with health outcomes. Contaminated sites require extensive cleanup efforts that can last months or even decades, that can strain a community’s patience and lead to frustration, social incohesion, and reduced well-being (Tucker, 1995). Well-being is a multidimensional concept which is shaped by expectations, beliefs, and experiences. It encompasses how people feel and function both on a personal and social level, and how they evaluate their lives as a whole (Jarden & Roache, 2023; Michaelson et al., 2012). Environmental factors such as access to green space, neighborhood safety, and air quality have been shown to influence well-being (Van De Weijer et al., 2022; Welsch, 2006).

Previous studies indicate that residents near contamination sites experience elevated levels of worry, stress, fear, and anxiety (Legg et al., 2023; McIntyre et al., 2018; Sullivan et al., 2021). However, little empirical evidence links proximity from a Superfund site with reduced well-being outcomes and more research is needed to identify specific risk factors for reduced well-being in communities affected by chronic environmental contamination. The majority of literature that has examined the psychological health outcomes as a result of living near a contaminated site have focused on pre-clinical or clinically diagnosed mental health conditions, such as depression and post-traumatic stress disorder that can be difficult to diagnose based on the waxing and waning nature of mental well-being over time, overlap of diagnostic criteria, and under-reporting (Chao et al., 2022; Legg et al., 2023; Schmitt et al., 2021). As a novel alternative to previous studies, the present study utilizes self-reported indictors of personal well-being.

### Theoretical Framework

Residential proximity (RP) to a source of environmental contamination may serve as a proxy for environmental risk perception. Environmental risk perception refers to an individual’s subjective judgment about environmental hazards, often based on limited and uncertain information and influenced by both the characteristics of the hazard and personal beliefs (Cortés et al., 2021; Liu et al., 2021). The primary residence is particularly meaningful, as it serves as the central location for daily life (Campbell et al., 1976) and represents a site of *rooting*, or spatial anchoring, reinforced by time, memories, and heritage (Kelly & Hosking, 2008). Several theoretical frameworks support the use of proximity to environmental contamination as an indicator of perceived risk, including Environmental Stress Theory (Lazarus and Cohen, 1977) the Psychometric Paradigm (Slovic, 1987), and the Protective Action Decision Model (Lindell & Perry, 2004).

The Environmental Stress Theory posits that stress arises when an individual perceives an environmental event as taxing or exceeding their ability to cope. This process is transactional, involving an appraisal of the event and an assessment of one's ability to manage it, leading to a stress response and the adoption of coping strategies (Lazarus & Cohen, 1977). Environmental conditions can interfere with optimal human functioning and are associated with four general types of stress identified by cognitive psychologists: cataclysmic events, stressful life events, daily hassles, and ambient stressors (Evans & Cohen, 1987).

Cataclysmic events include sudden disasters, both natural and technological, such as floods, earthquakes, and nuclear power plant accidents, exposing persons closest in proximity to the source of the hazard. Stressful life events involve major incidents that require personal or social adaptation within a defined timeframe, such as repairing property damage after a severe storm. Daily hassles encompass routine stressors that cause frustration, tension, or irritation, including noise and air pollution or increased industrial traffic in residential areas (Münzel et al., 2016; Roswall et al., 2015). Ambient stressors, in contrast, are stable, persistent environmental conditions that typically remain in the background but become noticeable when they interfere with personal goals or negatively impact health. For example, communities near Brownfield sites, which may be derelict, underutilized, or abandoned, often perceive these locations as potential pollution risks despite their inactive status, inhibiting future use and positing a threat to their health and environment (Wang et al., 2023).

The Psychometric Paradigm, developed by Slovic, Fischhoff, and Lichtenstein in the 1980s, offers a framework for evaluating subjective risk perception in a population that will predict a societal response such as to monitor the risky event, impose a regulation, or ban entirely (Slovic et al., 1985). The theoretical framework acknowledges that risk appraisals vary significantly among individuals and are influenced by risk characteristics such as voluntariness (e.g., imposed without consent), catastrophic potential (e.g., fatal consequences), and the degree of familiarity with a given hazard (Slovic et al., 1981, 1982; Slovic, 1987). The paradigm delineates the specific risk characteristics along two primary dimensions, “dread risk” and “unknown risk” and the authors note that the third factor tested, “societal and personal exposures” to the hazard, were found in their research to be correlated with perceived risk (Slovic et al., 1985).

Research within this paradigm has demonstrated that proximity to hazardous sources is frequently used as a surrogate for risk perception across disciplines, including environmental psychology, geography, and economics (Arias et al., 2017; Gayer et al., 2000; Maderthaner & Guttmann, 1978; Streimikiene, 2015). Researchers have reported that hazard proximity can influence risk perception as personal exposures to the environmental hazard are increased. A study by Gayer and colleagues (2000) reported that residential distance from a Superfund site was found to be correlated with health risks from the site, housing prices, and aesthetic disamenities (e.g., physical disorder, unattractive or perilous environmental settings).

Lastly, the Protective Action Decision Model (PADM), developed by Lindell and Perry (2004), provides insight into how individuals decide whether to respond to environmental hazards after being exposed to warning messages, environmental cues, and social cues (Lindell & Perry, 2004). This decision-making process depends on residents’ receipt, attention to, and comprehension of these messages (Lindell & Perry, 2012). An individual’s response can take the form of information search, protective action, or emotion-focused coping. In many cases, there is a feedback loop as additional cues or warnings are received. Information search occurs when individuals feel uncertainty at any stage of the process, which is common in chronic environmental contamination scenarios where the scope and severity of contamination are often unknown by both area residents and experts without lengthy investigations (Tucker, 1995).

The PADM model highlights that threat perceptions are influenced by factors such as geophysical vulnerability, ongoing concerns about personal and familial health, and heightened engagement with the hazard through environmental cues such as noise, sight, odor, traffic, and property devaluation or social cues that involve observing and interacting with concerned neighbors. One study that informed the development of the PADM examined the *perceived risk gradient*, which refers to how individuals weigh the risks and benefits of living near a nuclear facility (Lindell & Earle, 1983). The researchers found that this gradient is influenced by residential distance from the facility, shaping individuals’ perceived risks to health and safety. These early models suggest that closer proximity to a hazard increases interactions with the hazard source, making it a key factor in perceived risk and subsequent protective actions. In alignment with the Thomas theorem, residents'perception of the threat, rather than the objective risk, shapes their psychological and behavioral responses (Thomas & Thomas, 1928). Over time, these responses can impact personal well-being through prolonged or episodic stress reactions.

### The Present Study

The present study examines whether adults who lived near the Fernald Feed Materials Production Center (FMPC) Superfund site were a vulnerable population experiencing reduced personal well-being at the time of their enrollment in the Fernald Community Cohort (FCC), as measured by the Health Risk Appraisal (HRA). This study addresses an overlooked and understudied question in existing literature and aims to inform targeted remediation and community response strategies for future environmental disasters. Additionally, the study examines other potential risk factors for personal well-being in fully adjusted regression models, including demographic characteristics (e.g., age, sex, household income, and education level) and personal factors (e.g., self-reported chronic conditions and strength of social ties to family and friends).

This study explores the relationship between residential proximity (RP), used as a proxy for perceived environmental risk, and four well-being outcomes: life satisfaction, sense of control over life, life perspective, and overall physical health. These outcomes are measured using the HRA developed by the CDC in 1984 (Lasco et al., 1984). Data were drawn from 8,788 adults in the FCC, a population residing within a five-mile radius of the FMPC Superfund site in Ohio that operated as a uranium production facility from 1951 to 1989 (Wones et al., 2009). This study employs a cross-sectional design to test the hypothesis that participants who lived closer to the Superfund site at the time of cohort enrollment would report lower personal well-being compared to those who lived farther away, after adjusting for confounding factors in multivariable ordinal regression models.

## Material and Methods

### Study Participants

**Fig. 1:**
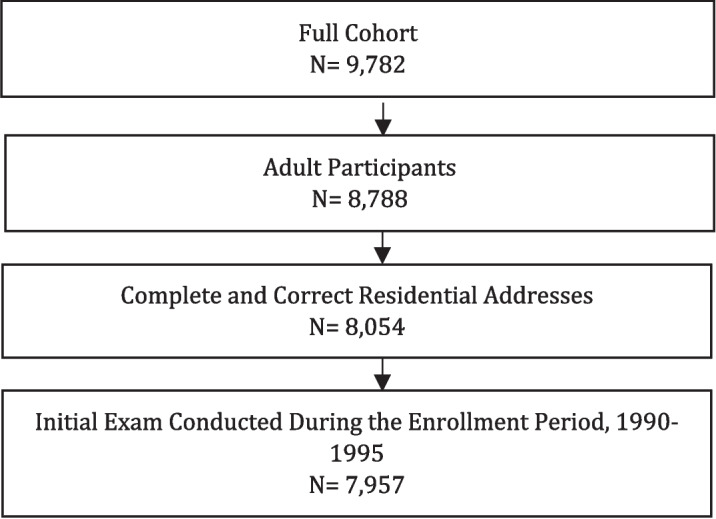
Flow chart of total study participants

The adult participants in the present study were drawn from a volunteer cohort of persons enrolled in a very large medical monitoring program to screen for and evaluate the health effects of the exposure to uranium because they lived within five miles of a uranium refinery. Enrollment began in 1990 and participants followed through 2008. Details of the FCC have been described extensively elsewhere (Pinney et al., 2003; Wones et al., 1995). The inclusion criteria for the present study included being an adult (aged ≥ 18 years) at the time of enrollment to the medical monitoring program, reporting complete and correct residential addresses that were verified for enrollment to the FCC, and completion of the baseline exam during the enrollment time period to the program. The present study focuses on participants who enrolled between January 1990 and December 1995, as only approximately 4% enrolled after 1995. Those who enrolled later may not adequately represent the effects of living near the FMPC borders on personal well-being, since they are temporally distant from the early cohort members. A flow chart of total participants included in the study is displayed (Fig. 1). The final sample size used in this analysis was 7,957 participants.

Informed consent was obtained from participants of the FCC at the time of each examination, including consent for their data to be used for research purposes and for study findings to be published. The program has full approval from the University of Cincinnati Institutional Review Board (IRB #: 2012–3745).

### Collection and Validation of Residential History

Residential history was collected via self-administered questionnaires during the first examination (Wones et al., 1995). Participants in the FCC listed all addresses where they had lived for at least three consecutive months within a five-mile radius of the FMPC between 1952 and 1984, including the start and end dates for each residence. At the second questionnaire, participants verified these addresses and dates.

To enhance reporting accuracy, a map showing concentric rings around the FMPC and local geographic features was used. Phone interviews were conducted with participants who did not return the second questionnaire or provided insufficient information. A random sample of addresses was verified using records from the Hamilton and Butler County Recorder of Deeds offices. All addresses were geocoded and categorized by concentric mile rings (0–5 mile radius) as a measure of RP, a surrogate for perceived environmental risk. The mile ring of the nearest residential address was used as the value for the RP variable, aligning with the theoretical framework guiding the research question: “Does proximity to a chronic environmental contamination site serve as a significant risk factor for reduced personal well-being?”.

### Health Risk Appraisal Tool

The measure of QoL for this study consisted of four items from the HRA. The HRA is a self-administered questionnaire originally designed to quantify a person’s chances of morbidity and mortality and to provide a target population for risk reduction interventions (Foxman & Edington, 1987). The original intent of the HRA questions were to calculate the risk of the participant dying within the following 10 years (Lasco et al., 1984). Over the years, many versions of the HRA have been developed since the original 1970 publication of “How to practice prospective medicine” manual by Robbins and Hall who had the intent to prospectively compute risk based on an established set of health risk criteria (Robbins & Hall, 1970). The HRA has been one of the most widely adopted tools in health education and health promotion programs (Yen et al., 2003). The HRA version used for the FCC was published by the CDC in 1980 and later modified by the University of Michigan in 1987. It used a 31-item, self-administered questionnaire to compute adult health risk (Gazmararian et al., 1991; Goetz et al., 1980). The estimated reliability of the HRA questions were found to have moderate to high test–retest correlations (*r* = 0.65–0.96) in a 1989 study by Smith and colleagues (Smith et al., 1989).

The assessment comprises various modifiable behavioral elements, such as smoking status, seat belt usage, fiber intake, and physical activity levels, measured on an ordinal scale. Lower scores indicate higher personal well-being for individual questions. The outcome variables of interest for this study are life satisfaction, life in control, life perspective, and overall physical health, reflecting participants' subjective feelings. Participants assess their life priorities, weighing positives and negatives to choose the most suitable response. Key questions from the assessment are detailed in the Supplementary Table in the Appendix. This comprehensive evaluation captures modifiable behaviors and subjective perceptions of well-being, providing valuable insights into individual health statuses.

### Physical Examinations

Participants of the medical monitoring program received comprehensive physical examinations every 2–3 years by a trained physician without regard to whether health conditions were potentially related to hazards from the FMPC (Wones et al., 2009). The FCC lasted for 18 consecutive years (1990–2008) however, for the purpose of this study, only the initial physical examination conducted on persons who enrolled between 1990 and 1995 was used in the cross-sectional analysis. The physical examination components included diagnostic and laboratory tests (see Wones & Pinney, 2009 for a full list of physical examination components) (Wones et al., 2009). In this study, systolic blood pressure, weight, and height records were adjusted for in the analysis.

### Outcome Measures

The outcome variables of interest from the HRA are life in control, life satisfaction, life perspective, and overall physical health status answer choices that are answered a Likert-style scale. The variable life in control has five possible answers: strongly agree, agree, neutral, disagree, and strongly disagree. The variable life satisfaction has four possible answer options: “completely satisfied, mostly satisfied, partly satisfied, and not satisfied”. Life perspective was first included in the HRA questionnaire later in the year 1991 and therefore, has fewer responses than the other variables (N = 5660). The response options are: “strongly agree, agree, neutral, disagree, and strongly disagree”. The last two response options for life in control and life perspective, “disagree and strongly disagree”, were combined in the analysis as one response category. The final outcome variable, overall physical health status, has four possible answers: “excellent, good, fair, and poor”.

### Covariates

The following variables from the physical assessment and HRA were chosen as potential confounders a priori to test in the model based on prior research rationale to be associated with well-being outcomes. All variables were measured at the time of the first examination that include age (y.), sex (male/female), self-reported chronic co-morbidities (“yes/no” variable derived from a self-report of cancer diagnosis, diabetes, heart disease, or chronic bronchitis/emphysema), perceived strength of social ties to friends and family (“not sure, weaker than average, about average, or very strong"), smoking pack years (y.), body mass index (BMI), systolic blood pressure (SBP, mmHg), household income (< $50,000/> $50,000), highest educational attainment (“some high school or less, high school graduate, some college, college graduate, vocational training, postgraduate or professional degree”), marital status (“single (never married), separated, married, divorced, widowed”), average alcohol consumption per week (drinks per week), average physical activity per week (“ < 1 time per week, 1 or 2 times per week, at least 3 times per week"), agreement with job satisfaction (“disagree strongly, disagree, agree/about average, strongly agree”), recent serious loss or misfortune (“no, yes- 1 loss or misfortune, yes- 2 or more serious losses in the previous year), and average hours of sleep per night (< 8 h/> 8 h).

### Statistical Analysis

Descriptive statistics were calculated for the participants’ demographic characteristics (Table 1). Continuous variables are expressed as means ± standard deviation of the mean and categorical variables are expressed as frequency and percent. Differences in these characteristics among the mile rings were observed using the overall Wald Chi-Square test for sociodemographic variables household income, marital status, and highest education level attained.

**Table 1:** Sociodemographic characteristics of the study participants by residential mile ring at the time of study enrollment, 1990–1995

Variable	n	0–1 Mile (*n* = 976)	1–2 Mile (*n* = 2646)	2–3 Mile (*n* = 1438)	3–4 Mile (*n* = 1400)	4–5 Mile (*n* = 1497)	Total (*n* = 7957)
Age (years, mean ± SD)	7957	43.5 ± 15	44.1 ± 14.6	44.1 ± 15.2	44.9 ± 15.6	45.9 ± 15.2	44.5 ± 14.9
Gender (n, %)	7957						
Female		532 (54.5)	1532 (57.9)	793 (55.2)	783 (55.9)	830 (55.4)	4470 (56.2)
Male		444 (45.5)	1114 (42.1)	645 (44.8)	617 (44.1)	667 (44.6)	3487 (43.8)
Income (n, %)*^ǂ^	7957						
> $50,000		226 (23.2)	600 (22.7)	409 (28.4)	419 (29.9)	435 (29.1)	2089 (26.2)
< $50,000		750 (76.8)	2046 (77.3)	1029 (71.6)	981 (70.1)	1062 (70.9)	5868 (73.8)
Marital Status (n, %)*ǂ	7781						
Married		681 (71.8)	1902 (73.4)	1020 (72.5)	1021 (74.6)	1086 (74.1)	5710 (73.4)
Single		135 (14.2)	326 (12.6)	217 (15.4)	213 (15.6)	197 (13.4)	1088 (14.0)
Separated/Divorced/Widowed		133 (14.0)	363 (14.0)	170 (12.1)	134 (9.8)	183 (12.5)	983 (12.6)
Education (n, %)*ǂ	7878						
College graduate and higher		157 (16.3)	369 (14.1)	311 (21.9)	375 (27.1)	299 (20.1)	1511 (19.2)
Some college or vocation		244 (25.3)	648 (24.7)	401 (28.2)	383 (27.7)	443 (29.9)	2119 (26.9)
High school graduate		385 (39.9)	1057 (40.3)	526 (37.0)	475 (34.3)	551 (37.1)	2994 (38.0)
Some high school or less		179 (18.5)	549 (20.9)	183 (12.9)	152 (10.9)	191 (12.9)	1254 (15.9)
* statistically different at *p* < 0.05 (comparisons between mile ring groups with the overall Chi-squared test) *n* number of participants, *SD* standard deviation of the mean ǂ at time of enrollment in the cohort							

As part of the quality control process, continuous data points, such as body mass index (BMI) and alcohol consumption, were examined as a potential outlier if they fell in the 99th percentile of distribution or were determined to outside of the “expected range” of values (Burcham et al., 2023). All predictor variables were assessed for missing data and the overall missing proportion was less than 5%. Multiple imputation approach was performed with ten imputed datasets for all variables except RP and the QoL outcome variables (Graham et al., 2007; Nguyen et al., 2021). Imputation was not performed on the QoL variables as the variables were missing at < 0.5%.

Fully adjusted logistic regression models were performed to assess the relationship between RP, a surrogate for perceived risk, and the dependent variables in separate models using ordinal logistic regression models as described by McCullagh in 1980 (McCullagh, 1980). The fully adjusted final model was chosen based on a forward model selection approach with forced entry with mile ring of the closest residential address to the Fernald FMPC borders, age, and sex with other significant variables added. All p-values in the logistic regression models are two-tailed and p-values < 0.05 were considered to indicate statistical significance. Interaction terms for residential group and age and sex were tested in each of the final models to determine if the combined effect of the demographic variables produced a significant effect. All data were analyzed using SAS Version 9.4 (SAS Institute Inc, 2013).

## Results

### Sample Participant Characteristics

Of the 8,788 adult participants in the medical monitoring program, 7,957, or 90.5%, had complete residential addresses on file. The majority of the participants were Non-Hispanic, White (99.5%). The average age of the participants was 44.5 y. ± 14.9 y. Females comprised 56.2% of the total sample population. The majority of the sample population reported a household income of less than $50,000 at the time of the initial questionnaire in 1990–1995 (73.8%), was married (73.4%), and were a high school graduate (38.0%) (Table 1).

The average BMI of the cohort was 27.5 ± 5.8, reported an average of 2.9 drinks of alcohol consumed per week ± 6.8, the mean cumulative smoking pack years of those who smoked was 8.5 y. ± 15.0 y., and the mean SBP was 129.6 mmHg ± 18.1 mmHg. The most commonly reported answers to key questions on the HRA among the full cohort of participants were awareness of environmental hazards “all or most of the time” (77.6%), a perception of “very strong” social ties in the community (48.2%), “about average” job satisfaction (48.4%), “no” recent losses or misfortunes in the past year (69.2%), “less than 8 h” of sleep, on average, per night (68.9%), and has never lived with a Fernald employee (91.3%).

**Fig. 2:**
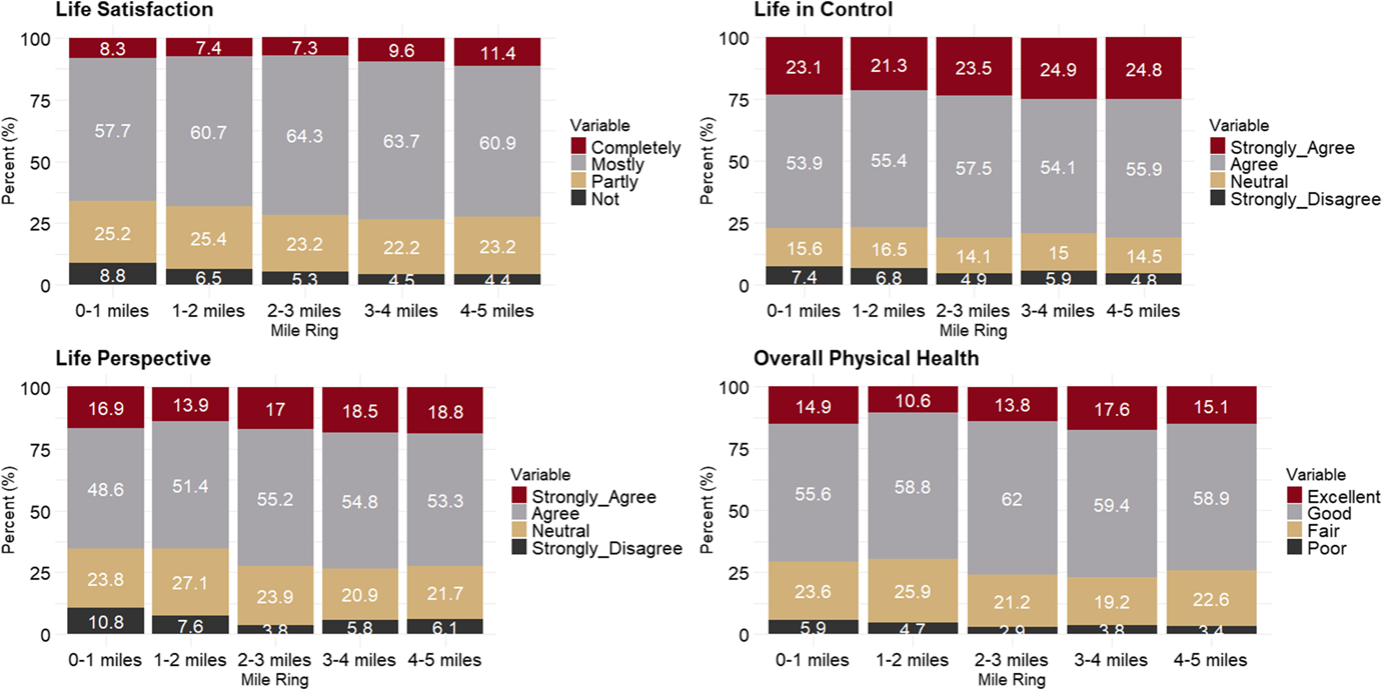
FCC participants’ unadjusted QoL responses by residential mile from the uranium processing facility at the time of enrollment

Participant responses to the HRA questions at the time of enrollment are summarized in Fig. 2 and Supplementary Table B. Most participants reported being “mostly” satisfied with their life (57.7–64.3%), “agreeing” that they had their life under control (53.9–57.5%) and in perspective (48.6–55.2%) and rated their overall physical health as “good” (55.6–62.0%) across all mile rings. These findings suggest that, despite living near a Superfund site, most participants reported high levels of personal well-being at the time of cohort enrollment. Since the HRA was administered upon enrollment only, participants may have felt optimistic about joining a rigorous medical monitoring program, which could have given them a sense of control over their health. Additionally, personal well-being likely fluctuated over time as new illnesses and chronic conditions emerged.

### Ordinal logistic regression analysis for QoL

Residential proximity to the FMPC was a significant risk factor for all well-being outcomes in the minimally adjusted models for age and sex. RP remained a significant factor for life satisfaction even after adjusting for additional demographic and lifestyle variables. In the fully adjusted logistic regression model, closer proximity to the contaminated site was associated with a more negative outlook on life satisfaction across different distance rings: closest mile ring [0–1 miles, OR 1.38, 95% CI: 1.17, 1.64], second closest mile ring [1–2 miles, OR 1.22, 95% CI: 1.07, 1.39], and third mile ring [3–4 miles, OR 1.19, 95% CI: 1.02, 1.38], compared to the furthest mile ring (4–5 miles). However, in fully adjusted models, RP (i.e., the closest residential address to the FMPC) was not significantly associated with life in control [OR 1.11, 95% CI 0.95–1.30], overall physical health [OR 1.03, 95% CI 0.87–1.20], or life perspective [OR 1.18, 95% CI 0.98–1.43] when comparing the closest residential group to the reference group (4–5 miles from the FMPC) (Table 2).

**Table 2:** Final fully adjusted logistic regression models examining residential distance from Fernald plant borders adjusted for significant co-variates in predicting reduced HRQoL outcomes

Predictor Variables	Model 1: Life Satisfaction	Model 2: Life in Control	Model 3: Overall Physical Health			Model 4: Life in Perspective
n	7939	7936	7938			5560
Max-rescaled R^2^	28.4%	20.7%	28.4%			25.6%
Predictor Variables	OR (95% CI)	OR (95% CI)	OR (95% CI)			OR (95% CI)
Mile Ring (ref: 4–5 miles)						
0–1 miles	1.38* (1.17, 1.64)	1.11 (0.95, 1.30)		1.03 (0.87, 1.20)	1.18 (0.98, 1.43)	
1–2 miles	1.22* (1.07, 1.39)	1.11 (0.98, 1.26)		1.11 (0.98, 1.26)	1.24 (0.70, 1.43)	
2–3 miles	1.19* (1.02, 1.38)	1.01 (0.88, 1.17)		1.11 (0.96, 1.28)	0.96 (0.82, 1.14)	
3–4 miles	0.98 (0.84, 1.15)	1.01 (0.88, 1.17)		1.01 (0.88, 1.17)	0.89 (0.75, 1.05)	
Age	0.99* (0.99, 1.00)	1.00* (0.99, 1.00)		1.00* (0.99, 1.00)	1.00* (0.99, 1.00)	
Sex (ref: male)	1.08 (0.98, 1.19)	1.09 (0.99, 1.20)		1.03 (0.94, 1.14)	1.04 (0.93, 1.15)	
BMI	–	–		1.01* (1.00, 1.02)	–	
Household income (ref: > $50,000)	1.25* (1.19, 1.39)	1.11* (1.00, 1.23)		1.09 (0.98, 1.21)	–	
Marital status (ref: married)	1.67* (1.50, 1.86)	–		–	–	
Education (ref: college and professional degrees)						
Some college or vocation	–	1.21* (1.06, 1.39)		1.14* (1.00, 1.30)	–	
High school graduate	–	1.44* (1.27, 1.63)		1.28* (1.13, 1.45)	–	
Some high school or less	–	1.56* (1.33, 1.83)		1.12 (0.95, 1.31)		
Cumulative smoking pack years	1.01* (1.00, 1.01)	1.00* (1.00, 1.01)		1.01* (1.00, 1.01)	–	
Recent loss or misfortune (ref: no)						
Yes, ≥ 2 losses	2.73* (2.37, 3.15)	1.72* (1.50, 1.97)		1.04 (0.90, 1.19)	2.79* (2.36, 3.32)	
Yes, 1 loss	1.77* (1.57, 2.00)	1.66* (1.48, 1.86)		1.08 (0.96, 1.21)	1.83* (1.61, 2.09)	
Hours of sleep (ref: > 8 h)	1.17* (1.05, 1.29)	–		1.06 (0.97, 1.17)	–	
Alcohol consumption	–	–		–	–	
Physical activity (ref: ≥ 3 times per week)						
1 or 2 times per week	1.15* (1.03, 1.29)	–		1.25* (1.12, 1.39)	–	
Less than 1 time per week	1.41* (1.26, 1.58)	–		1.39* (1.25, 1.54)	–	
Social ties (ref: very strong)						
About average	1.74* (1.58, 1.92)	1.85* (1.69, 2.04)		1.28* (1.17, 1.41)	2.13* (1.91, 2.38)	
Weaker than average/Not Sure	5.16* (4.33, 6.16)	4.28* (3.60, 5.07)		1.11 (0.93, 1.31)	6.58* (5.37, 8.06)	
Job satisfaction (ref: strongly agree)						
Agree/about average	3.16* (2.73, 3.66)	1.85* (1.69, 2.04)		1.37* (1.21, 1.55)	3.71* (3.15, 4.37)	
Disagree/strongly disagree	14.89* (12.41, 17.85)	9.50* (8.03, 11.24)		1.30* (1.11, 1.53)	11.08* (9.08, 13.53)	
Not employed	4.48* (3.77, 5.32)	3.35* (2.87, 3.92)		1.37* (1.18, 1.59)	4.99* (4.10, 6.07)	
Chronic co-morbidity (ref: no)	–	1.38* (1.21, 1.58)		1.03 (0.91, 1.18)	–	
Systolic Blood Pressure	–	–		0.99* (0.99, 1.00)	–	

^*^ statistically different at *p* < 0.05

*ref* reference

Response Levels:

Response Levels:

Model 1: Life Satisfaction: completely satisfied, mostly satisfied, partly satisfied, not satisfied

Model 2: Life in Control: strongly agree, agree, neutral, disagree/strongly disagree

Model 3: Overall Physical Health: excellent, good, fair, poor

Model 4: Life in Perspective: strongly agree, agree, neutral, disagree/strongly disagree

Notably, in the fully adjusted models, recent experiences of loss or misfortune, perceived strength of social ties, and job satisfaction emerged as having a significant association with well-being measures. For participants who reported two or more recent losses or misfortunes (n = 951, 12.0%), the odds of having a more negative life perspective is 2.79 times higher (95% CI: 2.36–3.32) compared to those who reported no recent losses or misfortunes (n = 5493, 69.2%), controlling for all other variables (see Table 2, Model 4). For participants who reported “weaker than average or not sure” about the strength of their social ties to family and friends (n = 608, 7.7%), the odds of having a more negative outlook on life satisfaction are 5.16 times higher (95% CI: 4.33–6.16) compared to those who reported "very strong" social ties (n = 3827, 48.2%), holding constant all other variables (see Table 2, Model 1). Interestingly, individuals who reported they “disagreed/strongly disagreed” with job satisfaction (n = 1217, 15.3%) had 14.89 times the odds of reporting a more negative outlook in life satisfaction [OR 14.89, 95% CI: 12.41—17.85] compared to those who reported “strong/very strong agreement” with job satisfaction (n = 1206, 15.2%) after adjusting for all other variables in the fully adjusted model.

An annual household income of less than $50,000 in 1990 to 1995 (n = 5868, 73.8%) was significantly associated with reduced well-being in two of the fully adjusted models: life satisfaction [OR 1.25, 95% CI 1.19–1.39] and life in control [OR 1.11, 95% CI 1.00–1.23] after holding all other variables constant. Additionally, cumulative smoking pack years at the time of study enrollment was marginally significant in three of the models: life satisfaction, life in control, and overall physical health and age was marginally significant in all four models. Other significant variables in the QoL models were the highest education level attained at the time of enrollment and reported hours of sleep per night.

Notably, alcohol consumption and self-reported chronic co-morbidities were not significant variables in any of the four QoL models. Systolic blood pressure was marginally significant in predicting overall physical health [OR 0.99, 95% CI 0.99–1.00]. Neither interaction terms for mile ring and age or mile ring and sex were statistically significant in any model. Interaction terms were tested between RP and age, sex, recent loss or misfortune, strength of social ties, and job satisfaction for each model, however, none were statistically significant.

### Life Satisfaction Model

The association between hazard source proximity and reduced life satisfaction in the fully adjusted model aligns with the study hypothesis and theoretical frameworks suggesting that proximity heightens awareness through environmental and social cues, warning messages, and direct interactions with hazards and therefore, participants living in close proximity would have higher risk of reduced personal well-being compared to residents further away from the hazard source. These factors contribute to the perceived threat of personal or societal harm. This perceived threat may negatively impact life satisfaction through underexplored biological pathways, including subjective risk appraisal and heightened, prolonged, or episodic stress responses (Chu et al., 2024). Additionally, the appraisal of personal well-being in the context of environmental contamination can be understood through psychological models that consider both emotional and cognitive dimensions (Das et al., 2020). Residents may experience negative emotions due to potential exposures that threaten their health, family, or property, while also cognitively evaluating these risks (e.g., cancer concerns) and engaging in harm-mitigation behaviors such as information-seeking. This aligns with the PADM, which describes common responses to environmental contamination when the scope and severity of exposure remain uncertain.

## Discussion

This study explores the relationship between RP to a chronic environmental contamination site and four well-being indicators from the HRA survey completed by Fernald community residents during their first examination (1990–1995). Using ordinal logistic regression, physical, sociodemographic, and psychosocial health variables were assessed. RP (≤ 2 miles) was significantly negatively associated with all well-being outcomes when minimally adjusted for age and sex and was linked to life satisfaction in fully adjusted models. Results showed that perceived social ties and job satisfaction were most commonly associated with the QoL outcomes.

Previous studies link RP to contaminated sites with decreased mental and physical health. A 2021 meta-analysis found that exposure to chronic environmental contamination sites were associated with anxiety, stress, depression, and PTSD (Schmitt et al., 2021). Sansom et al. reported that prolonged residence near hazardous substance facilities was associated with poorer self-rated physical health, adjusting for age, race, and sex (Sansom et al., 2017). In Cook County, Illinois, which has the highest landfill concentration per square mile in the U.S., White et al. noted that residents perceived landfills and hazardous waste disposal as significant community risks, with 43% rating their general health as "fair" or "poor" (White & Hall, 2015).

In these past studies, perceived social ties to family and friends emerged as protective against diminished well-being, consistent with previous research. Individuals with strong social ties, or "social capital," often exhibit higher self-efficacy, increased awareness, and a greater ability to effect change in their communities (Couch & Coles, 2011; Rook & Charles, 2017). This social capital serves as a valuable coping mechanism for stressors and fosters a sense of purpose in life (Umberson & Karas Montez, 2010). Job satisfaction was also significantly associated with life satisfaction and overall physical health (Unanue et al., 2017). Participants engaging in physical activities at least three times per week report higher levels of life satisfaction and better physical health ratings (Marquez et al., 2020). In the present study, strength of social ties, job satisfaction, and physical activity were significantly associated with well-being outcomes.

While subjective well-being isn't traditionally seen as a risk factor for chronic diseases, recent decades have seen a surge in well-being and quality of life research as health practitioners recognize its integral role in overall health status (Schulz et al., 2023). Self-assessed well-being impacts crucial biological pathways. For instance, studies show that psychological stressors such as natural disasters are linked to worse heart failure outcomes due to overactivation of the sympathetic nervous system (Harris et al., 2021). Environmental stressors near waste sites contribute to cumulative allostatic load (McEwen & Tucker, 2011). Psychological stress also increases cardiovascular disease risk via hormonal activation and inflammatory responses (Osborne et al., 2020). Residents near CEC sites may thus be vulnerable to both reduced well-being and long-term physiological outcomes that will be examined in future research within the FCC.

This study's strength lies in its use of multivariable ordinal logistic regression in a large cohort to examine the link between RP to the FMPC Superfund site and well-being outcomes. This is the only epidemiologic study of this scale to use the HRA to examine the relationship between residential proximity to chronic environmental contamination and personal well-being. The analysis extended to mile rings within five miles of the site borders, identifying risk factors for reduced well-being in a population chronically exposed to environmental contamination. The large sample size enabled the detection of subtle differences in effect size estimates during enrollment. Participants from the FCC received standardized HRA questionnaires and physical examinations, facilitating a thorough retrospective analysis of well-being.

An additional strength of the study is the use of subject-matter expertise on the history of the FCC to justify using the RP variable as a proxy for perceived risk of environmental exposure. This approach builds on qualitative interviews and historical knowledge of physical examinations and dosimetry project outcomes (Burcham et al., 2024). FCC residents were not informed of their uranium or radiation exposures, and population radiation estimates were published only in 1998 through a CDC-contracted dosimetry study, preventing bias in the RP used for this analysis (Killough et al., 1998). Individual exposure estimates have never been shared with members of the cohort. Public hearings indicated that materials processed at the facility were “heavy” and “didn’t come off the site,” which increased perceived exposure among nearby residents (Fernald Community Alliance, 2021). Previous studies, including those on the FCC population, suggest that a lack of trust in authorities and experts may have had the most significant impact on risk perception (Burcham et al., 2024; Gerhardstein & Brown, 2005; Wachinger et al., 2013).

### Limitations and Directions for Future Research

The study has several limitations. Firstly, the analysis did not encompass all nuanced personal, environmental, and job-related stressors experienced by the FCC community but focused on variables captured in the HRA. Research indicates that mental distress and feelings of loneliness mediate the relationship between adverse childhood experiences (ACEs) and well-being outcomes (Vederhus et al., 2021). The HRA did not include specific questions about ACEs, potentially accounting for unexplained variance in the models. Additionally, predictors of well-being identified in previous studies, such as emotional status, spirituality, self-esteem, time spent in leisure, and perception of physical safety, were not incorporated into this study (Borges et al., 2021; Patrício et al., 2014; Villas-Boas et al., 2019). The cross-sectional design provided insights into self-reported well-being at enrollment but restricted exploration of well-being changes in the cohort over time, longitudinal changes in the relationship between proximity and QoL, and precluded causal inferences. We would expect the cohort’s QoL to be negatively impacted over time as the cohort ages, chronic diseases develop (e.g., cancer diagnoses), and family members or loved ones become ill. Future research will leverage the 18 years of follow-up with the FCC, allowing for the analysis of changes to mental and physical well-being, health-related quality of life (HRQoL), and potential re-traumatization in response to environmental events. However, using baseline cohort data offers several advantages, including the largest sample size through the cohort follow-up period (N = 7,888), good generalizability to the source population living in the five-mile exposure domain from the Feed Materials Production Center (FMPC), and represents data collection closest in time to the adverse events of interest (e.g., knowledge of water contamination, class-action litigation, and media siege), which have been shown in previous studies to have a lasting negative impact on QoL.

It should be stated that the study population’s racial homogeneity limits exploration of race/ethnicity's impact on well-being, but it allows examination of health outcomes without the influence of environmental racism on personal well-being outcomes (Mascarenhas et al., 2021; Reuben et al., 2022). The predominantly White population of Fernald represents residents who lived within five miles of the FMPC site (Agency for Toxic Substances and Disease Registry et al., 1999). As previous studies have identified race/ethnicity as a non-modifiable risk factors for both the presence of a CEC site and reduced QoL, it is likely that under the similar conditions, reduced personal well-being may be observed in racially, ethnically, and economically diverse study populations, although we cannot make any assertions about these populations based on our present findings (Kim et al., 2023; Marshall, 2004; Mascarenhas et al., 2021). To enhance generalizability of the findings, future research should include residents living near CEC sites in other regions across the U.S.

### Public Health Implications

Our study makes significant contributions to understanding indicators of well-being and provides novel insights into risk factors associated with reduced well-being in communities affected by prolonged environmental contamination. Given the continued prevalence of Superfund sites in the U.S., our findings highlight that proximity to these sites may pose a substantial public health burden, making this research highly relevant for contemporary public health audiences.

Local health departments could benefit from incorporating validated QoL tools into community health assessments to identify regions requiring further investigation and support. This approach is particularly critical for communities impacted by EPA-designated Superfund sites, brownfields, and locations emitting toxic waste (Lodge et al., 2020; Wang et al., 2023). We also recommend that commercial industries undertake formal Environmental Impact Assessments, similar to those required of federal agencies under the National Environmental Policy Act of 1969 (Environmental Protection Agency, 2022). These assessments should include the identification of environmental stressors and their impacts on human health, including psychological well-being, within affected communities (Bhatia & Wernham, 2008).

Finally, recognizing the complexity and often sub-clinical nature of community-level QoL issues, local health departments should prioritize community awareness campaigns using material handouts, one-on-one interactions, referral resources, internet sites, and community meetings (Substance Abuse & Mental Health Services Administration, 2023). These campaigns can educate and empower residents to take proactive steps to enhance their individual well-being. Educational programs may focus on the health risks of living near contaminated sites and actionable strategies for improving personal health and well-being. Immediately initiating mental health resources at the community level, e.g., peer support groups, community counseling sessions, and mind–body wellness programs like therapeutic yoga and meditation classes, can help residents effectively cope with the challenges of living near these sites, particularly when geographic or social mobility and prior toxicant exposure cannot be altered (U.S. Agency for Toxic Substances and Disease Registry, 2025).

## Conclusions

In conclusion, we found notable differences in well-being outcomes associated with RP to a Superfund site. After accounting for model covariates, residents residing in the closest mile ring (0–1 miles) to the FMPC site border exhibited 38% higher odds of reporting reduced life satisfaction compared to those living in the reference mile ring 4–5. No significant differences in well-being were observed between mile ring 3–4 and 4–5 in any of the models. Notably, we found that perceived strength of social ties and agreement with job satisfaction were most commonly associated with the QoL outcomes of interest.

Our findings emphasize the importance of prioritizing the well-being of communities living in close proximity to an environmental contamination site. Additionally, we recommend future research that explores the relationship between self-rated health and physiological outcomes over time. This approach would provide valuable insights into the long-term impact of environmental exposures on both subjective and objective measures of well-being.

## Supplementary Information

Below is the link to the electronic supplementary material.

Supplementary file1 (DOCX 26 KB)
