# Supplementary material for: Living Near Contamination: The Impacts on Personal Well-Being
Source: Appl Res Qual Life. 2025 Jun 12;20(4):1419–41. doi: 10.1007/s11482-025-10456-8 (PMC12474628; doi:10.1007/s11482-025-10456-8)
Supplement: Supplementary file 1 — Supplementary file1 (DOCX 26 KB) [file 11482_2025_10456_MOESM1_ESM.docx]

**Supplementary Table A.** Key Health Risk Appraisal Variable Questions

| **Variable** | **Health Risk Appraisal Question** |
| --- | --- |
| **Outcome Variables** |  |
| Life Satisfaction | In general, how satisfied are you with your life? (Include personal and professional aspects) |
| Life in Perspective | Would you agree you have your life in perspective? |
| Life in Control | Concerning your daily life, would you agree that you have control over the day-to-day decisions affecting your function/performance? |
| Overall Physical Health | Considering your age, how would you describe your overall physical health? |
| **Predictor Variables** |  |
| Job Satisfaction | Would you agree you are satisfied with your job? |
| Social Ties | In general, how strong are your social ties with your family and friends? |
| Recent Loss or Misfortune | Have you suffered a personal loss or misfortune in the past year that had a serious impact on your life? |
| Hours of Sleep | How many hours of sleep do you usually get a night? |
| Weekly Physical Activity | On the average, how many times per week do you engage in physical activity, exercise, or work which increases the heart rate, causes you to breathe and sweat heavily, and is done at least 20 minutes in duration? |
| Weekly Alcohol Consumption | How many drinks of alcohol do you have in a typical week?  Bottles/cans of beer (12 oz)  Glasses of wine (6 oz)  Wine coolers (12 oz)  Mixed drinks/shots liquor (1.5 oz) |
| Environmental Hazards | Are you aware of potential safety and environmental hazards around you? |
| Lived with Fernald Employee | Have you ever lived with an employee of the Feed Materials Processing Plant, either a family member or a friend? |
| Chronic Co-morbidity | Do you have: heart problems, diabetes, cancer, or chronic bronchitis/emphysema? |

**Supplementary Table B.** Well-Being Outcomes by Residential Mile Ring at the Time of Enrollment.

| Variable | n | 0-1 Mile  (*n*=976) | 1-2 Mile  (*n* =2646) | 2-3 Mile  (*n* =1438) | 3-4 Mile  (*n* =1400) | 4-5 Mile  (*n* =1497) | Total  (*n* =7957) |  |
| --- | --- | --- | --- | --- | --- | --- | --- | --- |
| Life Satisfaction (n,%)* | 7939 |  |  |  |  |  |  |  |
| Completely satisfied |  | 81 (8.3) | 196 (7.4) | 104 (7.3) | 134 (9.6) | 170 (11.4) | 685 (8.6) |  |
| Mostly satisfied |  | 561 (57.7) | 1602 (60.7) | 920 (64.3) | 891 (63.7) | 910 (60.9) | 4884 (61.5) |  |
| Partly satisfied |  | 245 (25.2) | 671 (25.4) | 332 (23.2) | 310 (22.2) | 349 (23.3) | 1907 (24.0) |  |
| Not satisfied |  | 86 (8.8) | 172 (6.5) | 76 (5.3) | 63 (4.5) | 66 (4.4) | 463 (5.8) |  |
| Life in Control (n,%)* | 7936 |  |  |  |  |  |  |  |
| Strongly agree |  | 225 (23.1) | 563 (21.3) | 336 (23.5) | 349 (24.9) | 370 (24.8) | 1843 (23.2) |  |
| Agree |  | 524 (53.9) | 1463 (55.4) | 824 (57.5) | 756 (54.1) | 836 (55.9) | 4403 (55.5) |  |
| Neutral |  | 152 (15.6) | 434 (16.5) | 202 (14.1) | 210 (15.0) | 217 (14.5) | 1215 (15.3) |  |
| Disagree/ Strongly Disagree |  | 72 (7.4) | 179 (6.8) | 70 (4.9) | 82 (5.9) | 72 (4.8) | 475 (5.9) |  |
| Overall Physical Health (n,%)* | 7941 |  |  |  |  |  |  |  |
| Excellent |  | 146 (14.9) | 280 (10.6) | 198 (13.8) | 246 (17.6) | 226 (15.1) | 1096 (13.8) |  |
| Good |  | 542 (55.6) | 1553 (58.8) | 888 (62.0) | 832 (59.4) | 881 (58.9) | 4696 (59.1) |  |
| Fair |  | 230 (23.6) | 684 (25.9) | 304 (21.2) | 269 (19.2) | 337 (22.6) | 1824 (22.9) |  |
| Poor |  | 57 (5.9) | 123 (4.7) | 42 (2.9) | 53 (3.8) | 50 (3.4) | 325 (4.1) |  |
| Life in Perspective (n,%)* | 5560 |  |  |  |  |  |  |  |
| Strongly agree |  | 100 (16.9) | 253 (13.9) | 165 (17.0) | 180 (18.5) | 227 (18.8) | 925 (16.6) |  |
| Agree |  | 288 (48.6) | 935 (51.4) | 535 (55.2) | 533 (54.8) | 642 (53.3) | 2933 (52.8) |  |
| Neutral |  | 141 (23.8) | 494 (27.1) | 232 (23.9) | 204 (20.9) | 262 (21.7) | 1333 (23.9) |  |
| Disagree/ Strongly Disagree |  | 64 (10.8) | 138 (7.6) | 37 (3.8) | 56 (5.8) | 74 (6.1) | 369 (6.6) |  |
| *n* number of participants, *SD* standard deviation of the mean.  * statistically different at *p* <0.05 (comparisons between mile ring groups with the overall Chi-squared test) | | | | | | | | |
